# Morphology-based intraspecific taxonomy of Oreocryptophis porphyraceus (Cantor, 1839) in mainland China (Serpentes: Colubridae)

**DOI:** 10.24272/j.issn.2095-8137.2019.048

**Published:** 2019-07-18

**Authors:** Wang Ping, Shi Lei, Guo Peng

**Affiliations:** 1 College of Animal Science, Xinjiang Agricultural University, Urumqi Xinjiang 830052, China; 2 College of Life Science and Food Engineering, Yibin University, Yibin Sichuan 644007, China

**Keywords:** Taxonomy, Distribution, Morphology, Skull, Snake

## Abstract

In this study, a total of 106 individuals of *Oreocryptophis porphyraceus* from mainland China were morphologically examined and recorded. Differences between populations were compared by combining data from this study and other published research. The skulls of three specimens representing three proposed subspecies (i.e., *O. p. pulchra*, *O. p. vaillanti*, and *O. p. hainana*) were examined by computed tomography (CT) scanning. Both external morphological characters and skull comparisons consistently showed significant differences between the studied populations. Based on these data, we suggest that at least four subspecies of *O. porphyraceus* should be recognized in mainland China: i.e., *O. p. porphyraceus*, *O. p. pulchra*, *O. p. vaillanti*, and *O. p. hainana*. However, the taxonomical arrangement of the central Chinese populations with intermediate morphology remain unresolved.

The red-bamboo rat snake *Oreocryptophis porphyraceus* (Cantor, 1839) (Figure 1) is a medium-sized colubrid widely distributed in southern and southeastern Asia, including India, Nepal, Bhutan, Myanmar, Thailand, Vietnam, Laos, Malaysia, Singapore, Indonesia, and China (Boundy et al., 2014). Within China, the species ranges from southern Xizang in the west to Taiwan in the east and from Gansu and Shaanxi in the north to Hainan in the south (Zhao, 2006).

**Figure 1:**
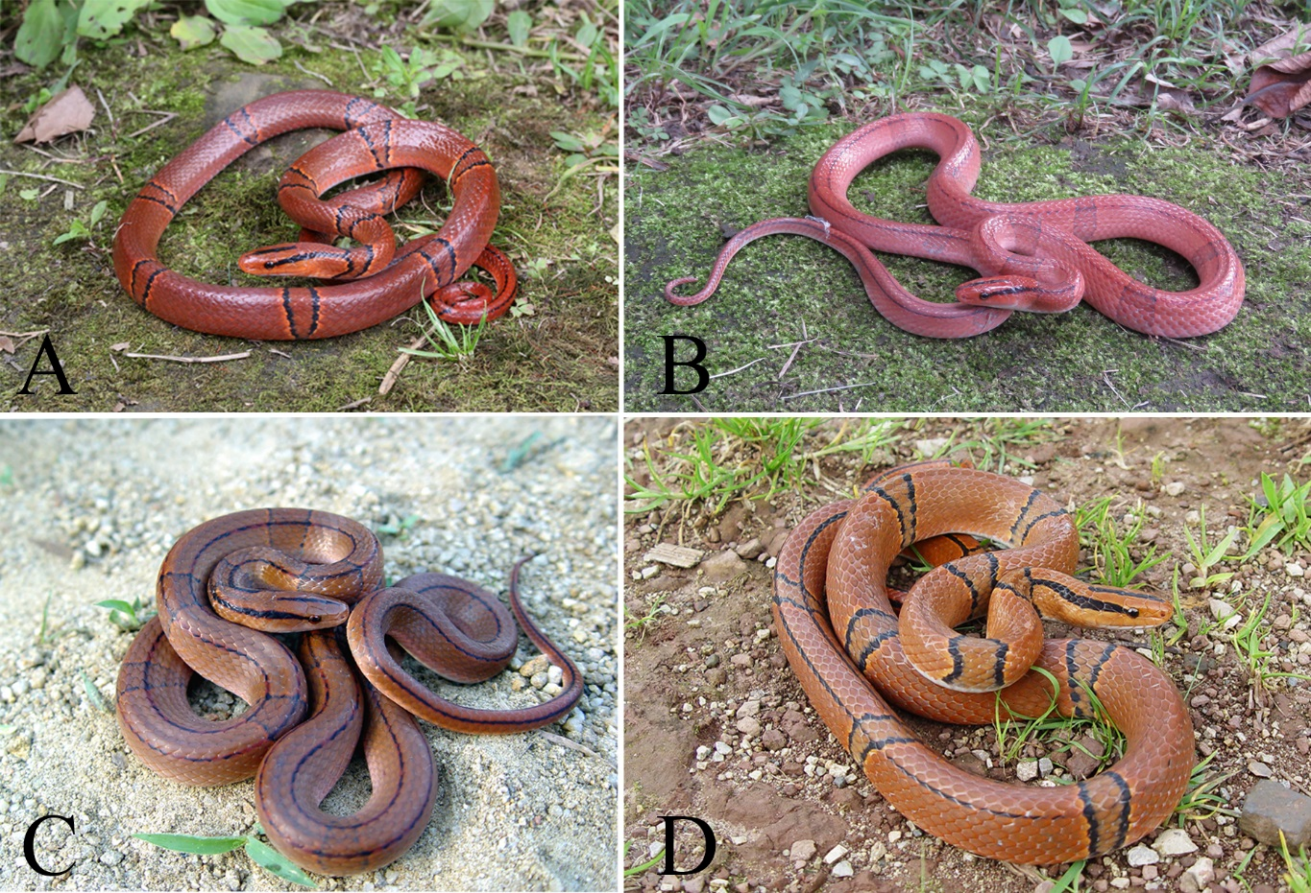
General view of *Oreocryptophis porphyraceus* from Yunnan (A: *O. p. pulchra*), Zhejiang (B: *O. p. vaillanti*), Hainan (C: *O. p. hainana*), and Sichuan (D: central China population)

The species was originally described as *Coluberporphyraceus* based on specimens from the Mishmee Hills (=Mishmii Hills) in Assam, India (Cantor, 1839), after which it was transferred into the genus *Elaphe* by Denburgh (1909). Finally, based on molecular phylogeny inferred from mitochondrial DNA, Utiger et al. (2002) erected the monotypic genus *Oreocryptophis* to accommodate the species.

The intraspecific taxonomy of *O. porphyraceus* has long been controversial. Nine subspecies have been proposed historically, with six originally described from China, though seven are thought to occur (Table 1).

**Table 1:** Subspecies proposed for *Oreocryptophis porphyraceus*

Subspecies	Original description	Type locality	Diagnostics	Distribution
*O. p. porphyraceus*	*Coluber porphyraceus* (Cantor, 1839)	Assam, India	Black flank stripe along posterior of body	India, Nepal, Bhutan, Myanmar, NW Thailand, W Yunnan China
*O. p. nigrofasciata*	*Psammophis nigrofasciatus* (Cantor, 1839)	Singapore (doubted), E China was designated as type locality by Smith (1930)	Black flank stripe along entire of body	E China, N Vietnam
*O. p. vaillanti*	*Simotes vaillanti* (Sauvage, 1877)	E China	Black head stripe not exceeding the posterior edge of parietal; black flank stripes along whole body	E China
*O. p. kawakamii*	*Liopeltis kawakamii* (Osima, 1910)	Taiwan, China	6th supralabial touching parietal, cross-bands 4 scales wide	Taiwan Province of China
*O. p. pulchra*	*Elaphe porphyracea pulchra* (Schmidt, 1925)	Kunming, Yunnan, China	Similar to *O. p. porphyracea*, but distinguished by fewer ventrals (177–185) and subcaudals (51–56)	Yunnan, Sichuan, W Guizhou, Gansu, Shaanxi of China
*O. p. hainana*	*Elaphe porphyracea hainana* (Mell, 1931)	Hainan, China	Similar to *O. p. nigrofasciata*, distinguished by more than 70 subcaudals	Hainan Province of China
*O. p. sikiangensis*	*Elaphe porphyracea sikiangensis* (Mell, 1931)	Luofushan, Guangdong, China	Similar to *O. p. nigrofasciata*, ventrals 195, subcaudals 72	Guangdong and Fujian provinces of China
*O. p. laticincta*	*Elaphe porphyracea laticincta* (Schulz, 1996)	Malaysia and Sumatra	Much wider cross-bands, covering 9–15 rows of dorsal scales	Malaysia and Indonesia
*O. p. coxi*	*Elaphe porphyracea coxi* (Schulz, 1996)	NE Thailand	Distinctly broad flank stripes, lacking cross-bands in adults, which may be observed on necks of juveniles	NE Thailand

Schulz & Entzeroth (1996) recognized seven of the nine subspecies mentioned in Table 1, except for *O. p. vaillanti* (Sauvage, 1877) and *O. p. sikiangensis* (Mell, 1931), based primarily on body patterns (Figure 2), and proposed that five subspecies, excluding *O. p. coxi* (Schulz & Entzeroth, 1996) and *O. p. laticincta* (Schulz & Entzeroth, 1996), could be found in China. Zhao (2006) suggested that *O. porphyraceus* should be identified as three subspecies in China: i.e., *O. p. porphyraceus* in southwest China, *O. p. nigrofasciata* (Cantor, 1839) in central and eastern China, and *O. p. hainana* (Mell, 1931) on Hainan Island. However, Das (2012) did not follow this taxonomical arrangement, instead advocating *O. p. pulchra* (Schmidt, 1925) in southern China, *O. p. vaillanti* in eastern China, and *O. p. kawakamii* (Oshima, 1910) on Taiwan Island. It should be noted, however, that all proposed taxonomical arrangements have been based on pholidosis, body patterns, and incomplete samples.

**Figure 2:**
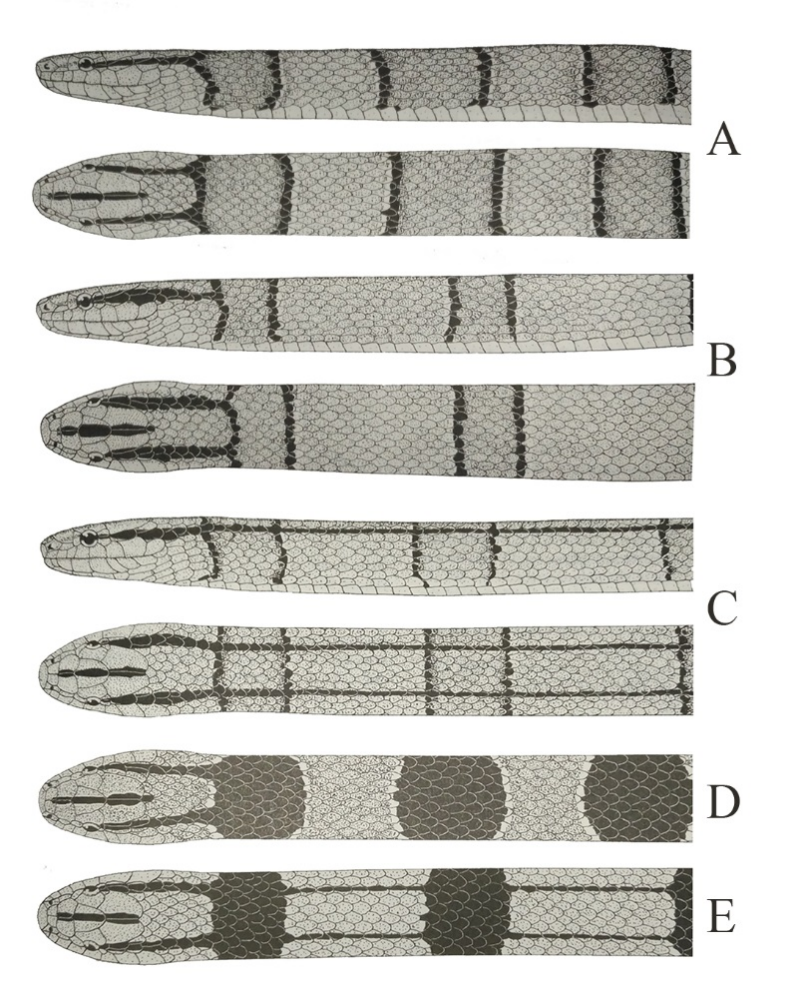
Body patterns of *Oreocryptophis porphyraceus* summarized by Schulz (1996) A: Adult *O. p. porphyraceus*; B: Adult *O. p. pulchra*; C: Adults *O. p. vaillanti* and *O. p. hainana*; D: Juveniles *O. p. porphyraceus* and *O. p. pulchra*; E: Juveniles *O. p. vaillanti* and *O. p. hainana*.

To explore the intraspecific diversity and clarify how many subspecies are present in mainland China, we examined the external morphology of *O. porphyraceus* specimens preserved in Chinese museums and also compared the skulls of several specimens from different populations.

In total, 47 characters related to scalation, coloration pattern, and body dimensions were examined and recorded for 106 specimens (49 males, 52 females, five juveniles; see Appendix I for details).

Measurements were taken with a digital slide-caliper to 0.1 mm except for snout-vent length (SVL), tail length (TL), and length of black flank stripes (BFS), which were measured by a measuring tape to 1 mm. The characters and their definitions are listed in Appendix II. Characters and their definitions followed Zhao (2006) and Zhong et al. (2017). For comparison, other data were obtained from previous literature (Pope, 1935; Schulz & Entzeroth, 1996; Zhao et al., 1998).

The skulls of three specimens from Yunnan (YBU 14076), Zhejiang (YBU 17246), and Hainan (YBU 12007), representing three proposed subspecies (i.e., *O. p. pulchra*, *O. p. vaillanti*, and *O. p. hainana*), were examined using CT scanning at the Chengdu Institute of Biology (CIB), Chinese Academy of Sciences (CAS). Specimens were scanned at 70 KV with a flux of 114 μA, with other parameters set following Shi et al. (2017). A total of 720 transmission images were reconstructed into a 2 048×2 048 matrix of 802 slices using VGStudio max (a three-dimensional reconstruction program) developed by CIB, CAS. Based on the photos, 20 characters were recorded or measured by direct counting, Snake Measure Tool software, or digital slide-caliper. Measurements and descriptive methods for different bones and characters followed Cundall (1981) and Guo et al. (2010) and their abbreviations are listed in Appendix II.

Among the 106 specimens examined, the maximum total length was 1 096 (930+166) mm. The following characters were identified: 9–16 cross-bands on body, 2–5 on tail; each cross-band occupying 2–17 rows of vertebrae. Loreal 1 (absent in three individuals); preocular 1; postoculars 2; temporals 1+2 (rarely 1+1, 1+3); supralabials 8, 3-2-3 (rarely 7, 3-2-2, 2-2-3); infralabials 8–10, 3-2-3, 3-2-4, 4-2-3, or 4-2-4 (rarely 11, 4-2-5), with first 4–5 touching anterior chin-shields; dorsal scales in 19-19-17 rows (rarely 19-19-15), all smooth; ventrals 177–203 in males, 181–209 in females; subcaudals 53–76 pairs in males, 50–71 in females. Two black flank stripes extending from tip of tail through to whole body or interrupted at mid body. Black stripe present on middle of head, sometimes extending beyond posterior edge of parietal; on lateral head, two black stripes extending immediately behind eyes to first cross-band or to black flank stripes.

Several characters were significantly different between populations. For example, the specimens from southwestern China (Yunnan, Western Guizhou, and Southwestern Sichuan, same below) showed black head stripe exceeding posterior edge of parietal (vs. absent in other populations) and black flank stripes not exceeding half of total length (vs. exceeding half of total length in other populations); specimens from the Hainan population possessed more than 70 pairs of subcaudals (vs. less than 70 pairs in other populations). A detailed comparison of external morphology is listed in Supplementary Table S1.

The skulls of the three representative specimens were generally consistent with other colubrid snakes (Cundall, 1981; Zhang, 1988). The skulls were also phenotypically similar in some characters, including bulbiform parietal and post-orbits not touching frontals (Figure 3). However, the skulls also exhibited several differences; e.g., weak parietal ridge in southwestern China populations (vs. strong in other populations), posterior margin of frontal straight in Hainan population (vs. curved in other populations), supratemporals extending beyond posterior end of braincase in other populations (vs. not in southwestern China population), post-orbits triangular in southwestern China population (vs. broad T-shaped in eastern inland China population and arc-shaped in Hainan population). Detailed descriptions and comparisons of the three skulls are listed in Supplementary Table S2.

**Figure 3:**
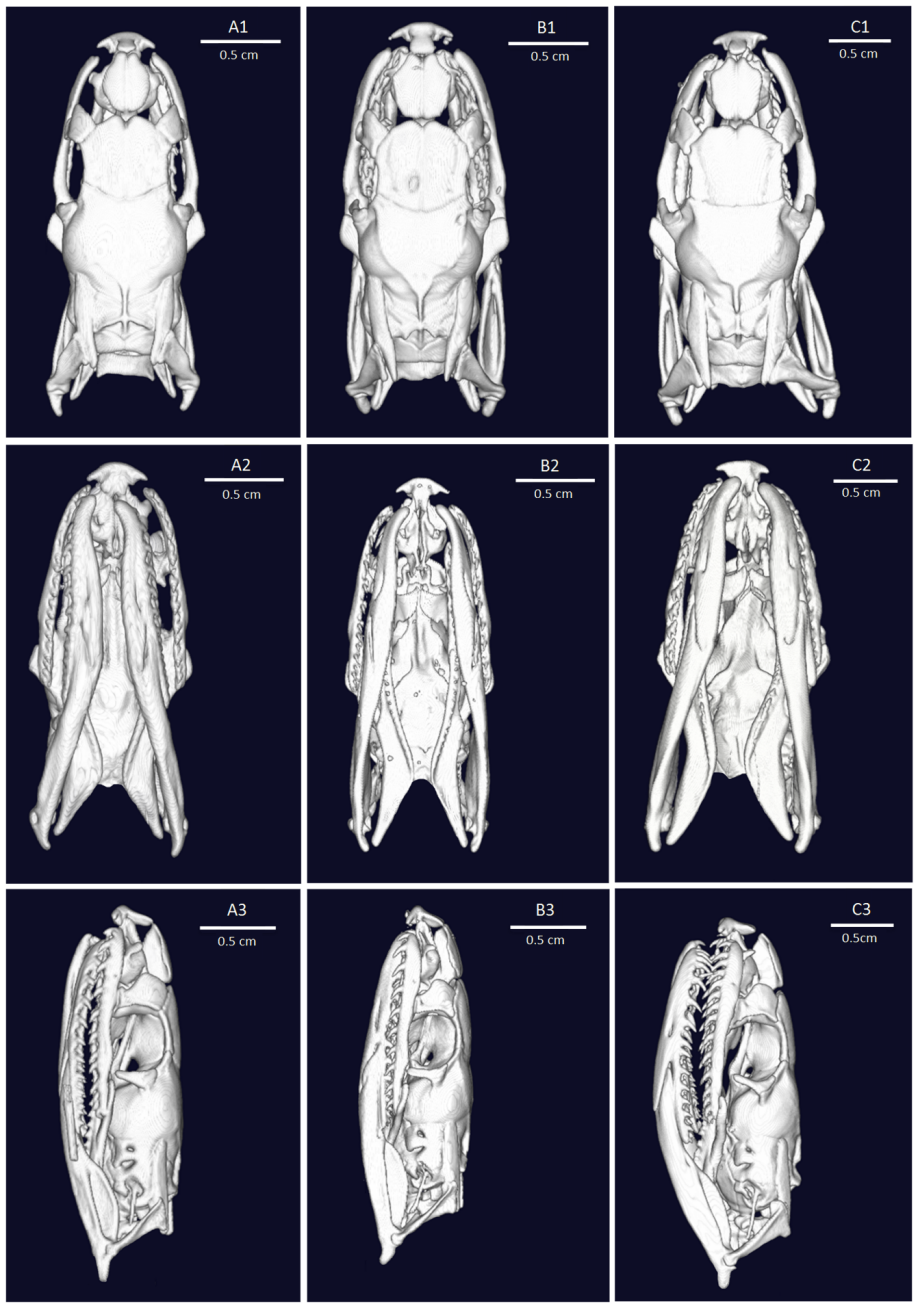
CT scans of skulls of *Oreocryptophis porphyraceus* from Yunnan (A: *O. p. pulchra*), Zhejiang (B: *O. p. vaillanti*), and Hainan (C: *O. p. hainana*)

Based on comparison of external morphology and skull characteristics, we suggest that at least four subspecies of *O. porphyraceus* should be recognized in mainland China: i.e., *O. p. porphyraceus*, *O. p. pulchra*, *O. p. vaillanti*, and *O. p. hainana*. The diagnostic characters and approximate geographical distribution of each subspecies are discussed below and shown in Supplementary Table S3.

*Oreocryptophis p. porphyraceus* (Figure 2 A) was originally diagnosed by “bright porphyry-red, with black transverse lines edged with white, posterior portion of body with two black parallel dorsal lines; beneath light yellow; ventral scales 213; subcaudals 64” (Cantor, 1839). No topotype was available for morphological examination in this study. However, Prof. Jing Che from the Kunming Institute of Zoology, Chinese Academy of Sciences, recorded a juvenile (KIZ019349) from Motuo county, Xizang Autonomous Region, China, which is very close to the type locality of this subspecies (personal communication). The specimen possessed 206 ventrals, 58 pairs of subcaudals, and 23 cross-bands on its body, much more than that in other populations. Considering its proximity to the type locality, we regard the specimens from southeastern Xizang as *O. p. porphyraceus*.

*Oreocryptophis p. pulchra* was named based on specimens from Kunming, Yunnan Province. It was distinguished from *O. p. porphyraceus* by having fewer ventrals (177–185) and subcaudals (51–56 pairs) (Schmidt, 1925).

Morphological comparison indicated that specimens from the southwestern China population shared the following characters: ventrals less than 209, cross-bands on body 11–17, black head stripe extending beyond posterior edge of parietal, and black flank stripes visible only on posterior third of body (Figures 1A, 2B; Supplementary Table S1). Additionally, the skull from Yunnan was slender, with very weak parietal ridge, supratemporals did not exceed beyond posterior edge of braincase, and post process of prefrontal was not significant (Figure 3A; Supplementary Table S2). The numbers of ventrals and subcaudals fell within the range of those from the southern Yunnan, western Guizhou, and southwestern Sichuan populations (177–209 ventrals and 50–67 subcaudals, respectively. Supplementary Table S1).

Based on the above, we agree with Das (2012), Schmidt (1925), and Schulz & Entzeroth (1996) that the specimens from southwest China, including Yunnan, western Guizhou, and southwestern Sichuan should be identified as *O. p. pulchra*.

Three names have been proposed for the eastern inland China (Jiangsu, Anhui, Zhejiang, Jiangxi, Fujian, northeastern Guangdong, Hong Kong, eastern Hunan, eastern Hubei, and southeastern Henan) population: i.e., *Psammophis nigrofasciatus* (*O. p. nigrofasciata*), *Simotes vaillanti* (*O. p. vaillanti*), and *Elaphe porphyracea sikiangensis* (*O. p. sikiangensis*). *Oreocryptophis p. nigrofasciata* was originally described based on specimens from Singapore and diagnosed by “light reddish-yellow above, with broad cross-bands and two barbed dorsal lines of the same color; the interval between these dorsal lines dotted with black; ventrals 245; subcaudals 75” (Cantor, 1839). Smith (1930), followed by several other authors (e.g., Pope, 1935; Schulz & Entzeroth, 1996; Zhao, 2006), suggested that the type locality of *O. p. nigrofasciata* was most likely incorrect, and thus eastern China was designated as the type locality of this subspecies. Within the specimens (40 individuals) we examined from eastern inland China, the maximum number of ventrals was 209 (214 in Zhao (2006)), much lower than that in the original description (Cantor, 1839); in addition, there were no more than 70 pairs of subcaudals. Obviously, the specimens from eastern inland China could not be identified as *O. p. nigrofasciata*.

The specimens from eastern inland China shared the following characters: cross-bands on body less than 11, black head stripe not exceeding posterior edge of parietal, black flank stripes extending along whole body, less than 70 pairs of subcaudals (Figures 1B, 2C; Supplementary Table S1). From the Zhejiang specimen skull, the anterior portion was short, ventral process of basioccipital was strong, and posterior edge of supraoccipital was strong (Figure 3B; Supplementary Table S2). These characters were distinct from the other populations but were congruent with the diagnostic characters of *O. p. vaillanti* proposed by Sauvage (1877): “The black head stripe does not exceed the posterior edge of the parietal, the black flank stripes extend from the back of the eyes and along the body to the end of the tail”. *Oreocryptophis p. sikiangensis* was originally described from Guangdong Province, China, as by having 195 ventrals and 72 subcaudals (Mell, 1931). In fact, except for the subcaudals, its pholidosis and color pattern are in line with those of *O. p. vaillanti*. Thus, we suggest that the population from eastern inland China should be recognized as *O. p. vaillanti*.

The specimens from Hainan Island were originally described as *Elaphe porphyracea hainana* (*O. p. hainana*) and as having more than 70 pairs of subcaudals (vs. less than 70 in other subspecies). This arrangement was accepted by Schulz & Entzeroth (1996), but not by Das (2012); Zhao et al. (1998) proposed that some specimens from Guangdong and Guangxi have more than 70 pairs of subcaudals, and thus stated that this subspecies was likely invalid. However, Zhao (2006) subsequently recognized the validity of *O. p. hainana*.

Based on our examination and previously published data (Zhao et al., 1998), 17 out of 19 specimens from Hainan (89.5%) had more than 70 subcaudals, with only two females (10.5%) having fewer (Supplementary Table S1). The specimens from Hainan also exhibited the following unique skull characters: blunt, basioccipital process tall, posterior end of frontal straight, and post-orbit arc-shaped (Figure 3C; Supplementary Table S2).

Therefore, based on external and skull morphology, we suggest that *O. p.hainana* is valid, and maybe endemic to Hainan, China.

In addition to the populations mentioned above, the central China population (including northern Guangxi, eastern Guizhou, western Hunan, Chongqing, northern and eastern Sichuan, Gansu, Shaanxi, western Henan, and western Hubei) exhibited intermediate external morphology between the southwestern China and eastern inland China populations in body coloration and pholidosis. For example, the average numbers of ventrals, subcaudals, and cross-bands (189.83/58.8/10.68) of the central China population were between those of the southwestern China population (185.36/56.65/13.52) and eastern inland China population (196.06/64.61/9.8); the black flank stripes extended along the whole body or were only present in the posterior part of the body (Supplementary Table S1); and occasionally the black flank stripes were indistinct or intermittent anteriorly. However, based on current data, we cannot conclude to which subspecies it should be assigned, or whether the central China population represents a different subspecies altogether.

It should be noted that the above taxonomical arrangement was mainly based on morphological comparison. Subspecies boundaries and particularly subspecies geographical distributions were not clearly determined. Further study using genetic data and complete sampling could provide evidence to clarify these issues.

## APPENDIX I

Information for specimens examined in this study.

CCNU: Central China Normal University; CIB: Chengdu Institute of Biology, Chinese Academy of Sciences; FJNU: Fujian Normal University; GZNU: Guizhou Normal University; HNNU: Hainan Normal University; KIZ: Kunming Institute of Zoology, Chinese Academy of Sciences; SYSU: Sun Yat-Sen University; YBU: Yibin University.

Chongan, Fujian: CIB9308–9310, CIB9312–9314, CIB9316–9318, CIB64I5354, FJNU3571008, FJNU3571011, FJNU3571012, FJNU3571014, FJNU3571015, FJNU3571018–3571020, FJNU3571023, FJNU3571024, FJNU3571026, FJNU3571028. Fuzhou, Fujian: FJNU3571022, FJNU3571030. Putian, Fujian: FJNU3571021, FJNU3571035, FJNU3571036. Nanping, Fujian: KIZ056354–056356. Dongyang, Zhejiang: YBU17273, YBU17246. Shangrao, Jiangxi: SYSU000646, SYSU000665. Shaoguan, Guangdong: SYSU000821. Hainan: HNNUR0008, HNNUR0268, HNNUR1017, YBU12007, CIB9328. Chengzhou, Hunan: CIB9329–9331. Nanning, Guangxi: CIB9319. Guilin, Guangxi: SYSU000234, KIZ750003. Baise, Guangxi: YBU11202. Wuzhou, Guangxi: YBU17252. Fangchenggang, Guangxi: YBU15138. Suizhou, Hubei: CCNU0011753. Huanggang, Hubei: CCNU0000022. Yichang, Hubei: YBU13322A. Kunming, Yunnan: CIB78020, CIB9297, CIB9298, CIB9302, CIB94021, KIZ640015, KIZ640016, KIZ640008, KIZ83004, KIZ87001, KIZ87003. Pu’er, Yunnan: CIB78019, KIZ75II0202, KIZ75II0195, KIZ75II0840. Honghe, Yunnan: KIZ85I0069, YBU14076, KIZ85I0660. Baoshan, Yunnan: CIB9303–9306, CIB00358. Xishuangbanna, Yunnan: CIB9299, CIB9300, KIZ75008, KIZ75I360, KIZ75I411, KIZ75I430. Mianyang, Sichuan: CIB9294, CIB94011, CIB102877. Leshan, Sichuan: CIB9290, CIB9292, CIB9295. Pengzhou, Sichuan: CIB9296, CIB83610, CIB83611. Luzhou, Sichuan: YBU071076. Chongqing: CIB9293, CIB9307. Leishan, Guizhou: CIB03363, CIB9320–9322, CIB9324, CIB9325. Weining, Guizhou: CIB9327. Rongjiang, Guizhou: CIB99428, GZNUGS0056. Jiangkou, Guizhou: YBU13204. Liuan, Anhui: CIB9332.

## APPENDIX II

Characters recorded and their abbreviations.

External morphology: SVL: Snout-vent length; TL: Tail length; Vs: Number of ventrals; Sc: Number of subcaudals; RBFSL: Ratio of black flank stripes to total body length; NCB: Number of cross-bands; BHS: Black head stripe exceeding posterior edge of parietal or not; CBCRD: Cross-band on body covering dorsal rows along body vertebrae; CTCRD: Cross-band on tail covering rows of dorsal tail scales along caudal vertebrae; CBBL: Length of cross-band on body along body vertebrae; CBTL: Length of cross-band on tail along caudal vertebrae; SIL: Length of sulcus between internasals; SPfL: Length of sulcus between prefrontals; FL: Length of frontal; HL: Length of head; ML: Length of mouth; SupOL: Length of supraoculars; PL: Length of parietal; NL: Length of nasals; LL: Length of loreals; BFSL: Length of black flank stripes; MWI: Maximum width between outside of internasals; MWPf: Maximum width between outside of prefrontals; FW: Maximum width of frontal; HW: Maximum width of head; PW: Maximum width of parietals; SupOW: Maximum width of supraoculars; NW: Width of nasals; LW: Width of loreals; EO: Diameter of eyes; BHSL: Length of black head stripe; CBW: Position where sides of cross-bands transversely extend; VS19–17: Ventral position corresponding to reduction from 19 to 17 scale rows of dorsal; Sc12–10: Subcaudal position corresponding to reduction of dorsal tail scales from 12 to 10 scale rows; Sc10–8: Subcaudal position corresponding to reduction of dorsal tail scales from 10 to 8 scale rows; Sc8–6: Subcaudal position corresponding to reduction of dorsal tail scales from 8 to 6 scale rows; Sc6–4: Subcaudal position corresponding to reduction of dorsal tail scales from 6 to 4 scale rows; Sc4–2: Subcaudal position corresponding to reduction of dorsal tail scales from 4 to 2 scale rows.

Skull bones: An: Angular; Bo: Basioccipital; Bs: Basisphenoid; Co: Columellar; Cp: Compound bone; Dt: Dentary; Ec: Ectopterygoid; Eo: Exoccipital; Fr: Frontal; Mx: Maxilla; Na: Nasal; Pa: Parietal; Pro: Pre-orbit; Pl: Palatine; Pm: Premaxilla; Po: Post-orbit; Pr: Prootic; Pt: Pterygoid; Qt: Quadrae; Sm: Septomaxilla; So: Supraoccipital; Sp: Splenial; St: Supratemporal; Vo: Vomer.

Skull characters: SL: Skull length; SW: Skull width; AP: Anterior portion; PL: Parietal length; FL: Frontal length; StL: Supratemporal length; PoL: Postfrontal length; SPLW: Skull proportion of SL (from front top of premaxilla to post end of exoccipital along mid line of head) to SW (widest parietal); RAPS: Relative length of anterior portion to HL; RLFr: Relative length of frontal to HL; RLP: Relative length of parietal to HL; RLS: Relative length of supratemporal to HL; RLQ: Relative length of quadrate to HL; RLM: Relative length of mandible to HL; RLPf: Relative length of postfrontal to HL; NPaT: Number of palatine teeth; NMT: Number of maxilla teeth; NDT: Number of dentary teeth; PR: Parietal ridge; SEBB: Supratemporal extending beyond posterior end of braincase; FSE: Fused (or not) supraoccipital and exoccipital; BoP: Basioccipital process; PP: Prefrontal process; BsP: Basisphenoid process; PeF: Posterior end of frontal; SdP: Supraoccipital dorsal process; SPo: Shape of post-orbit.
